# Novel assembly strategy cracks open the mysteries of walnut genome evolution

**DOI:** 10.1038/s41438-019-0143-5

**Published:** 2019-04-05

**Authors:** G. Albert Wu, Fred G. Gmitter

**Affiliations:** 1 US Department of Energy Joint Genome Institute, Walnut Creek, CA USA; 2 Citrus Research and Education Center (CREC), Institute of Food and Agricultural Sciences(IFAS), University of Florida, Lake Alfred, FL USA

**Keywords:** Plant evolution, Genome

## Abstract

High quality chromosome-scale assemblies from an interspecific hybrid between walnut and a wild relative reveal the persistence of asymmetric fractionation between the sub-genomes and suggest a late-Miocene origin for the genus *Juglans*.

Persian or English Walnut (*Juglans regia)*, a diploid species (2*n* = 32) native to the mountainous regions of Central Asia, is an important nut crop worldwide. The genus *Juglans* comprises about 25 species divided into four sections, with the Old and New World distributions^1^. A high-quality reference genome sequence of walnut is of great value for walnut breeding and providing insights into some fundamental biological questions (Fig. 1). Existing assemblies for several species of *Juglans* have provided important insight into the genetic diversity of the genus, as well as walnut domestication^2,3^. However, the fragmentation of existing assemblies has limited their application.

**Fig. 1:**
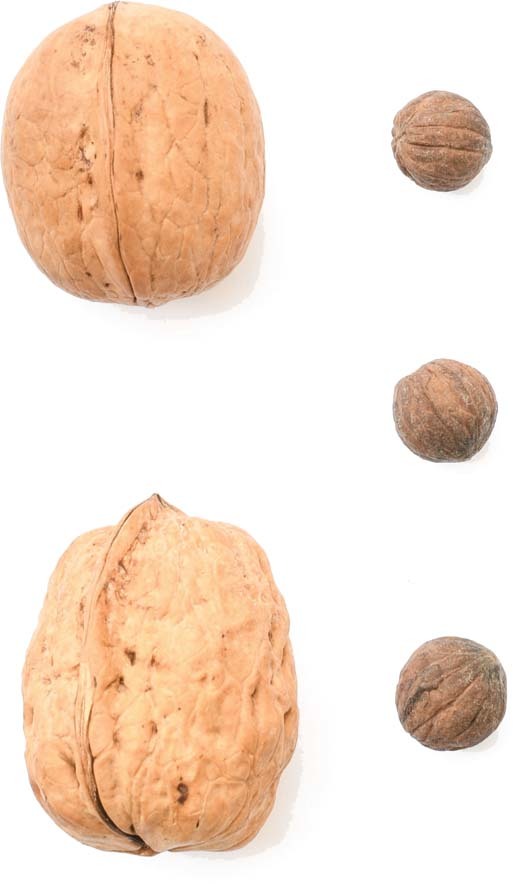
Images of nuts of *J. regia* (left) and *J. microcarpa* (right) native to Asia and North America, respectively

The first chromosome-scale high quality reference sequence for walnut is now available resulting from the use of a combination of long-read sequencing and optical genome mapping technologies^4^. The authors report a novel and cost-effective strategy of sequencing, assembling and phasing an interspecific hybrid (*J. microcarpa x J. regia*), which circumvented the difficulty of assembling highly heterozygous genomes. Both parents of the hybrid are wind-pollinated obligate outcrossing plants characterised by highly heterozygous genomes. The deep divergence between the two parental species (Ks = 0.03) and PacBio long read sequencing technology, and mapping the assembled sequences of the hybrid onto optical maps of the parental species, made it possible to allocate sequences of the hybrid to the parental genomes.

The final genome assemblies contain 535 Mb and 528 Mb of sequence for English walnut (*J*. regia) and its wild relative (*J. microcarpa*), respectively, with more than 99% of the sequences anchored to 16 chromosomes of each species. Genome annotation resulted in 31,425 and 29,496 protein-coding genes for walnut and *J. microcarpa*, respectively, with transposable elements accounting for 41% of the genome. The assembly is highly contiguous with scaffold N50 = 34.8 Mb, which was limited by the length of chromosomes. By comparison, a previous walnut genome assembly^3^ (*J. regia* cv Chandler) based on Illumina sequencing has an N50 scaffold size of 640 kb. The high-quality assembly also reveals the structure and mechanism of forging telomeres, as well as the evolution and dynamics of centromere repositioning, thereby relating these phenomena to the growing body of evidence across the plant kingdom.

The genus *Juglans* (Juglandaceae) went through a whole-genome duplication (Juglandoid WGD) around the Cretaceous-Paleogene (K-Pg) boundary 66 MYA^5^ and the eight pairs of homoeologous chromosomes manifest a highly asymmetric fractionation, with 18,179 and 13,107 genes located on the dominant and subdominant subgenomes of *J. regia,* respectively. The difference in gene loss was shown to be nearly constant among different homoeologous chromosome pairs and the rate of gene loss is approximately uniform along chromosomes, except in centromeric regions where a higher rate of gene loss was observed. The *J. regia* and *J. microcarpa* assemblies also afforded the authors an opportunity to examine the differential gene loss between the two subgenomes into the more recent past, i.e., since the divergence of *J. regia* and *J. microcarpa* approximately 8 MYA. The observed persistence of this asymmetric fractionation since the Juglandoid whole genome duplication suggests that the causal factor is still active in the *Juglans* genomes. The authors attributed this active factor to asymmetric gene expression, as genes on the dominant subgenome were, on average, more highly expressed relative to their paralogues on the subdominant genome. Further, they suggest further study of methylation of specific LTR-RTs and corresponding siRNAs to determine whether the mechanism underlying the observed reduced gene expression is the same as observed in *Arabidopsis* and *Brassica*^6^.

The timing of the Juglandoid WGD and sequence divergence between paralogous genes in the *Juglans* genomes can be used to estimate the molecular clock rates. Based on over six thousand pairs of paralogues in the *J. regia* genome, the authors estimated the genome-wide sequence divergence to be Ks = 0.33 and Ka = 0.074, corresponding to nucleotide substitution rates of 2.5 × 10^−9^ per synonymous site per year and 5.6 × 10^−10^ per nonsynonymous site per year, respectively. Assuming a constant molecular clock, these rates imply that *J. regia* and *J. microcarpa* diverged in the late Miocene approximately 6–8 MYA, which is much younger than some previous estimates^1,3,7^ but much older than others^8^. The authors provided other lines of evidence in support of the late-Miocene diversification of *Juglans*, including the divergence times of *Amborella* and grape, and the origin of the gamma whole genome triplication, in addition to the Juglandaceae fossil record.

Finally, because of the potential importance of *J. microcarpa* × *J. regia* hybrids as disease resistant rootstocks useful for commercial production of walnut, the authors catalogued the R genes (resistance gene analogues) in both species into 11 classes, and compared their numbers and distributions. Having this resource available is an important step of practical value in efforts focused on genetic improvement and development of new walnut rootstocks.
